# Postbiotic_Loaded Hyalurosome: Investigating Their Potential as Antimicrobial and Antioxidant Agents

**DOI:** 10.1111/jocd.70777

**Published:** 2026-04-18

**Authors:** Seyede Fateme Morakabi, Mahvash Khodabandeh Shahraky, Gholamreza Ahmadian, Roghayeh Mokhtari Asl

**Affiliations:** ^1^ National Institute of Genetic Engineering and Biotechnology Tehran Iran

**Keywords:** antibacterial, antioxidant, hyalurosomes, nanoparticles, postbiotics, probiotics, skin microbiota

## Abstract

**Background:**

The skin microbiota supports skin health by defending against pathogens and regulating immunity, while its imbalance can lead to disorders. With rising interest in natural ingredients, postbiotics (bioactive metabolites from probiotic fermentation) are emerging as natural antimicrobial and antioxidant agents.

**Aims:**

This study evaluates the antibacterial and antioxidant properties of cell‐free supernatants (CFS) from four lactic acid bacteria (LAB) (*Lacticaseibacillus rhamnosus* [*LGG*], *Limosilactobacillus reuteri* [*LR*], *Limosilactobacillus fermentum* [*LF*], and *Weizmannia coagulans* [*WCO*]) and encapsulates the two most potent samples (one with the highest antibacterial and the other with the strongest antioxidant activity) into hyaluronic acid–based nanoparticles (hyalurosome) for enhanced topical delivery.

**Methods:**

Growth curve analysis, supernatant preparation, antibacterial assays (determination of minimum inhibitory concentration [MIC], minimum bactericidal concentration [MBC], and inhibition zone), antioxidant assay (measuring the scavenging activities of 2,2‐diphenyl‐1‐picrylhydrazyl [DPPH assay]), nanoparticle synthesis and characterization, and cytotoxicity testing were performed.

**Results:**

*LGG* demonstrated potent, broad‐spectrum antibacterial activity, showing the highest efficacy against *Micrococcus luteus* ( *M. luteus* ) (30 mm inhibition zone, MIC/MBC of 5/7.5% v/v). In contrast, *WCO* exhibited superior antioxidant capacity, with an IC₅₀ of 17.48 μg/mL in the DPPH assay, outperforming ascorbic acid (IC₅₀ = 32.50 μg/mL). The optimized hyalurosomes were spherical, monodisperse (80–200 nm, PDI < 0.7), and retained the bioactivity of the postbiotics. *LGG*‐hyalurosomes achieved complete eradication of *M. luteus* and *Staphylococcus aureus* ( *S. aureus* ) within 48 h, demonstrating sustained release, while *WCO*‐hyalurosomes maintained strong antioxidant activity (IC₅₀ = 37.63 μg/mL). Crucially, encapsulation significantly reduced cytotoxicity, increasing human dermal fibroblast viability from 60% (free CFS) to over 90% at a 6.25% (v/v) concentration.

**Conclusion:**

These results position LAB‐derived postbiotics delivered via hyalurosomes as a potent and safe dual‐function strategy, addressing both microbial dysbiosis and oxidative stress in advanced skincare.

## 1 Introduction

Human skin acts as a sophisticated biological shield, constantly interacting with the external environment while maintaining an internal balance [1]. Beyond its structural and protective roles, the skin hosts a diverse array of microorganisms, collectively known as skin microbiota, which are crucial for immune regulation, pathogen defense, and maintaining skin equilibrium [2].

Increasing evidence suggests that disruptions in this delicate microbial ecosystem are closely linked to various skin disorders, including acne, atopic dermatitis, eczema, and premature aging. Under these circumstances, the skin microbiome changes and, in certain cases, plays a role in the pathophysiology of disease [3, 4].

The study of the skin microbiome has become a promising area with the potential to create groundbreaking solutions for maintaining skin health and treating skin diseases [5]. These days, there are numerous methods to modulate the microbiota arrangement to enhance the host's health. Microbiome‐based therapies, which have garnered significant interest in cosmetic dermatology, are an emerging class of live biotherapeutic products with significant potential as a new intervention platform for skin disorders. In addition to using established wild‐type strains that naturally occur on the skin, these therapies can be modified to produce specific therapeutic molecules, enabling more customized treatment options [4, 6].

New microbiome‐based processes, such as fecal microbiota transplant, probiotics, and phage therapy, are being invented. Furthermore, metabolites derived from probiotics have been observed to have a significant potential to control diseases affecting the derm. Although live probiotics have been explored as a means of restoring microbial balance, their instability, short shelf life, and formulation challenges have limited their widespread topical use [7, 8]. This has shifted focus toward postbiotics—nonviable microbial cells, cell fractions, or metabolites that confer physiological benefits without requiring bacterial viability [9]. Postbiotics derived from lactic acid bacteria (LAB), particularly *Lactobacillus* species, exhibit significant antimicrobial, anti‐inflammatory, and antioxidant properties, making them valuable for skin health and rejuvenation [10].

However, a major challenge in using postbiotics for topical treatment is ensuring effective delivery across the skin barrier without compromising their bioactivity [11]. Traditional formulations often struggle to maintain stability or provide controlled release of active ingredients. To address these challenges, nanotechnology‐based delivery systems have been developed, offering enhanced bioavailability, sustained release, and reduced irritation potential of the drugs [12]. Among the available nanocarriers, hyaluronic acid (HA), a naturally occurring glycosaminoglycan abundant in the extracellular matrix, has gained significant interest because of its excellent biocompatibility, moisture‐retaining ability, and inherent antiaging benefits [13]. Recent advancements in HA‐based nanocarriers, such as hyalurosomes, have demonstrated improved encapsulation efficiency and targeted delivery of bioactives, suggesting that HA nanoparticles could serve as an ideal platform for incorporating postbiotics into modern skincare products [14].

We hypothesize that encapsulating LAB‐derived postbiotics within hyalurosomes will enhance their stability, skin permeability, and controlled release, thereby amplifying their inherent antimicrobial and antioxidant efficacy. To test this, the present study: (1) optimizes hyalurosome fabrication parameters, (2) characterizes their physicochemical properties, (3) evaluates their antibacterial and antioxidant activities in vitro, (4) assesses their biocompatibility with human dermal fibroblasts, and (5) quantifies their skin permeation efficiency. By integrating the bioactivity of postbiotics with the delivery advantages of HA‐based nanotechnology, this work aims to develop a novel strategy for addressing skin microbiota dysbiosis and oxidative stress.

## 2 Materials and Methods

### 2.1 Material

- *Lacticaseibacillus rhamnosus ATCC 53103, Limosilactobacillus reuteri ATCC 23272, Limosilactobacillus fermentum ATCC 9338, Weizmannia coagulans ATCC 5856, Escherichia coli ATCC 25922, Staphylococcus aureus ATCC 65389, Micrococcus luteus ATCC 10240* (National Institute of Genetic Engineering and Biotechnology, NIGEB).
- Culture media: de Man, Rogosa, and Sharpe (MRS) broth, Mueller Hinton broth (MHB) (Ibresco Life Science).
- Chemicals: 3‐(4,5‐dimethylthiazol‐2‐yl)‐2,5‐diphenyltetrazolium bromide (MTT), 2,2‐diphenyl‐1‐picrylhydrazyl (DPPH), ascorbic acid, hyaluronic acid (HA, 50 kDa), gentamicin sulfate, penicillin–streptomycin (Pen‐Strep), lecithin, agar, chloroform, methanol, dimethyl sulfoxide (DMSO) (Sigma‐Aldrich; Merck KGaA; Neutron).
- Buffers and solutions: Phosphate‐buffered saline (PBS), fetal bovine serum (FBS) (BioIdea).
- Cell line: Human dermal fibroblasts (continuous cell line).

### 2.2 Isolation of Cell‐Free Supernatants (CFS) From Lactic Acid Bacteria

Cell‐free supernatants were produced [15] from cultures of *LGG, LR, LF*, and *WCO*.

- Bacterial growth: Bacterial strains were cultivated in MRS broth (0.1% v/v) at 37°C for 17 h.
- CFS preparation: Following incubation, the bacterial cultures were further incubated in fresh MRS broth at 37°C overnight under static conditions. Subsequently, the bacterial cells were removed by centrifugation at 6000 rpm for 10 min at 4°C.
- CFS concentration: The resulting CFS was concentrated tenfold by lyophilization and subsequently sterilized using a 0.22‐μm filter (Membrane Solutions, USA).

### 2.3 Bactericidal Effects of CFS From Lactic Acid Bacteria

#### 2.3.1 Antimicrobial Susceptibility Testing via Well Diffusion Assay

The antimicrobial activity of tenfold concentrated CFS produced from *LGG, LR, LF*, and *WCO* was investigated against three bacterial strains: *S. aureus* (Gram‐positive), *M. luteus* (Gram‐positive to Gram‐variable), and *E. coli* (Gram‐negative).

A well‐diffusion assay was used to assess the antimicrobial activity [16]. Briefly, MH agar plates were inoculated with 1% of each bacterial strain (OD at 600 nm = 0.3). Wells with a diameter of 8 mm were created using sterile 200 μL pipette tips. Subsequently, 50 μL of each tenfold concentrated CFS, 4 μg/mL gentamicin (positive control), or PBS (negative control) was added to the respective wells. The plates were then incubated at 37°C for 16 h. The zones of inhibition surrounding each well were measured to determine the antimicrobial activity.

#### 2.3.2 Determination of Minimum Inhibitory Concentration (MIC) and Minimum Bactericidal Concentration (MBC)

The MICs of tenfold CFS from LAB against *E. coli, S. aureus*, and *M. luteus* were determined using a broth microdilution method adapted from the Clinical and Laboratory Standards Institute (CLSI) guidelines [15].

A 96‐well microtiter plate was used to perform serial two‐fold dilutions of the CFS in Mueller‐Hinton (MH) broth, resulting in a concentration range of 50% to 0.39%. Additional intermediate concentrations (40% to 0.31% and 30% to 0.20%) were included to increase assay resolution.

To determine the MIC, 0.1 mL of each CFS dilution was added to wells containing 0.1 mL MH broth. Subsequently, 10 μL of each bacterial suspension (adjusted to 0.5 McFarland turbidity) was added to each well, except for the control.

Bacteria‐free medium served as the negative control, and antibacterial‐free medium inoculated with bacteria served as the positive control. The microplates were incubated at 37°C for 24 h.

After incubation, the plates were gently shaken, and the optical density (OD) at 600 nm was measured using an ELISA reader. The MIC was defined as the lowest concentration of CFS that inhibited visible bacterial growth.

To determine the MBC, 30 μL aliquots were taken from three wells: the last turbid well and the two subsequent clear wells. The aliquots were then plated onto MH agar plates and incubated. MBC was defined as the lowest concentration of CFS that resulted in a > 99.9% reduction in bacterial viability.

### 2.4 Evaluation of Antioxidant Activity: DPPH Radical Scavenging

The antioxidant capacity of the LAB CFS was assessed [17] by measuring its ability to scavenge stable 1,1‐diphenyl‐2‐picrylhydrazyl (DPPH) radicals. The assay was adapted from the method described by Lin and Chang [18]. Briefly, 100 μL of ethanolic DPPH solution (7.88 mg/100 mL) was combined with 25 μL of the respective LAB CFS in a 96‐well microplate. The mixture was vigorously mixed and incubated in the dark for 30 min at room temperature (25°C ± 2°C). Subsequently, the absorbance of each well was measured at 517 nm using a microplate reader. The percentage of DPPH radical scavenging activity was calculated using the following equation:

$$\%\text{DPPH Radical Scavenging}=\left[\left(A_{\text{control}}-A_{\text{sample}}\right)/A_{\text{control}}\right]\;x\;100$$

where the control represents the absorbance of the DPPH solution without the sample and the sample represents the absorbance of the reaction mixture containing the LAB CFS.

### 2.5 Hyalurosome Preparation

**FIGURE 1:**
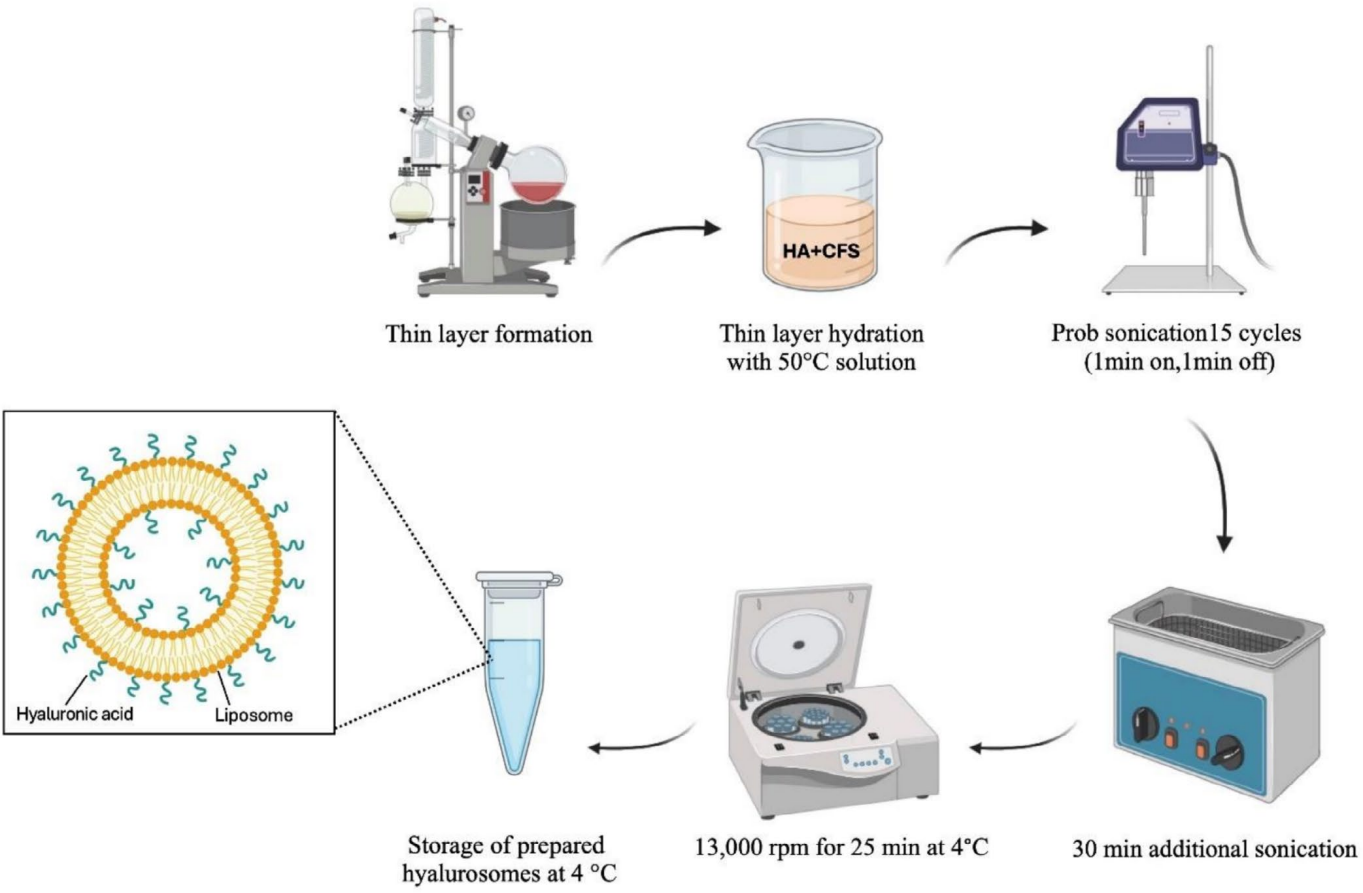
Schematic illustration of hyalurosome preparation by the modified film hydration and extrusion method. The lipid (Lecithin) thin film was formed using a rotary evaporator and subsequently hydrated with a hyaluronic acid (HA) and tenfold cell‐free supernatant (CFS) of LGG/WCO at 50°C. The resulting dispersion was subjected to probe sonication for 15 cycles (1 min on/1 min off). The suspension was then incubated in a water bath for 30 min and centrifuged at 13 000 rpm for 25 min at 4°C.

Hyalurosomes were prepared using a modified film hydration and extrusion method [19]. Lecithin (50 mg) was dissolved in 1 mL of chloroform in a round‐bottom flask. The solvent was subsequently removed using a rotary evaporator under reduced pressure (80 mbar) at 50°C for 30 min to form a thin lipid film. The dried lipid film was then hydrated with 10 mL of a solution containing 5 mL of a tenfold CFS of LGG/WCO and 5 mL of hyaluronic acid (HA) solution (5 mg/mL). The resulting suspension was sonicated for 15 min. Further sonication was performed using an ultrasonic probe for a total of 15 cycles (1 min sonication followed by 1 min rest) in an ice bath. The suspension was then sonicated for an additional 30 min and subsequently centrifuged at 13000 rpm for 25 min at 4°C (Figure 1). The supernatant was collected and the prepared hyalurosomes were stored at 4°C for further use.

### 2.6 Characterization of Hyalurosomes

#### 2.6.1 Hyalurosome Morphology

The formation and morphology of hyalurosomes were investigated using scanning electron microscopy (SEM). To prepare samples for SEM analysis, a 1:10 dilution in distilled water was performed. Subsequently, the samples were dried at 37°C and coated with a thin layer of gold (10 nm) under vacuum conditions.

#### 2.6.2 Particle Size and ζ Potential

The size, polydispersity index (PDI), and ζ potential of hyalurosomes were determined by the dynamic light scattering (DLS) technique.

#### 2.6.3 Evaluation of the Release Kinetics of *LGG* ‐Hyalurosome by Normal Saline Test (CFU Assay Was Used)

In order to check the antibacterial properties of *LGG*‐hyalurosome, a colony count test was designed with the normal saline method [20]. For this assay, briefly, 500 μL of *LGG‐hyalurosome* were added to an Erlenmeyer containing 4 mL saline solution (0.9% NaCl in DI) and 500 μL of *M. luteus* (OD = 0.1) and incubated at 37°C with a shaker of 180 rpm.

*LGG* unloaded hyalurosome were added to 4.5 mL of saline solution and 500 μL of *M. luteus* as a positive control under the same conditions.

At 24 and 48 h, samples were taken from each of the Erlens. Then, the diluted solution inside each Erlenmeyer flask was cultured on a solid plate containing MH agar and was incubated overnight at 37°C. The number of colonies that appeared on the plates after incubation was counted and evaluated for the determination of antibacterial efficiency of *LGG*‐hyalurosome against *M. luteus* .

#### 2.6.4 Evaluation of Encapsulation of *WCO* ‐Hyalurosome by DPPH Assay

The DPPH radical scavenging activity of the tenfold CFS of *WCO*‐hyalurosome was determined using the method described in part 2.5. Briefly, 100 μL of DPPH solution (7.88 mg/mL in ethanol) was mixed with 25 μL of the diluted CFS in a 96‐well microplate. The mixture was incubated at room temperature in the dark, and absorbance at 517 nm was measured at 0, 24, and 48 h using a microplate reader.

### 2.7 MTT Assay for Cytotoxicity Evaluation

The cytotoxicity of free and encapsulated postbiotics (*LGG* and *WCO*) was evaluated on human dermal fibroblasts using the 3‐(4,5‐dimethylthiazol‐2‐yl)‐2,5‐diphenyltetrazolium bromide (MTT) assay [21]. Human dermal fibroblasts were seeded at a density of 10 000 cells/well in 96‐well microplates and incubated for 24 h (37°C, 5% CO₂) to achieve 70%–80% confluency. The culture medium was then replaced with fresh medium containing serial dilutions (50% to 6.25% v/v) of CFS or hyalurosome‐encapsulated postbiotics. Control wells received untreated medium or medium alone (blank). Following 24 h of incubation, 30 μL of MTT solution (5 mg/mL in PBS) was added to each well, and plates were incubated for 4 h (37°C, dark) to allow formazan crystal formation. The MTT solution was aspirated, and 100 μL of dimethyl sulfoxide (DMSO) was added to dissolve the crystals. After 30 min of agitation (200 rpm), absorbance was measured at 570 nm using a microplate reader (BioTek Synergy H1). Cell viability (%) was calculated as [(*A*_treatment_ − *A*_blank_)/(*A*_control_ − *A*_blank_) × 100%], where *A* represents untreated cells.

### 2.8 Statistical Analyses

Data are expressed as mean ± standard deviation (SD) of triplicate experiments. Statistical significance was determined using one‐way analysis of variance (ANOVA) followed by Tukey's post hoc test for pairwise comparisons (GraphPad Prism v9.0, GraphPad Software, USA). Factors analyzed included treatment type (free CFS vs. encapsulated postbiotics), concentration (50% to 6.25% v/v), and bacterial strain (*LGG* vs. *WCO*). A *p*‐value < 0.05 was considered statistically significant.

## 3 Results and Discussion

### 3.1 The Growth Kinetic of Lactic Acid Bacteria

**FIGURE 2:**
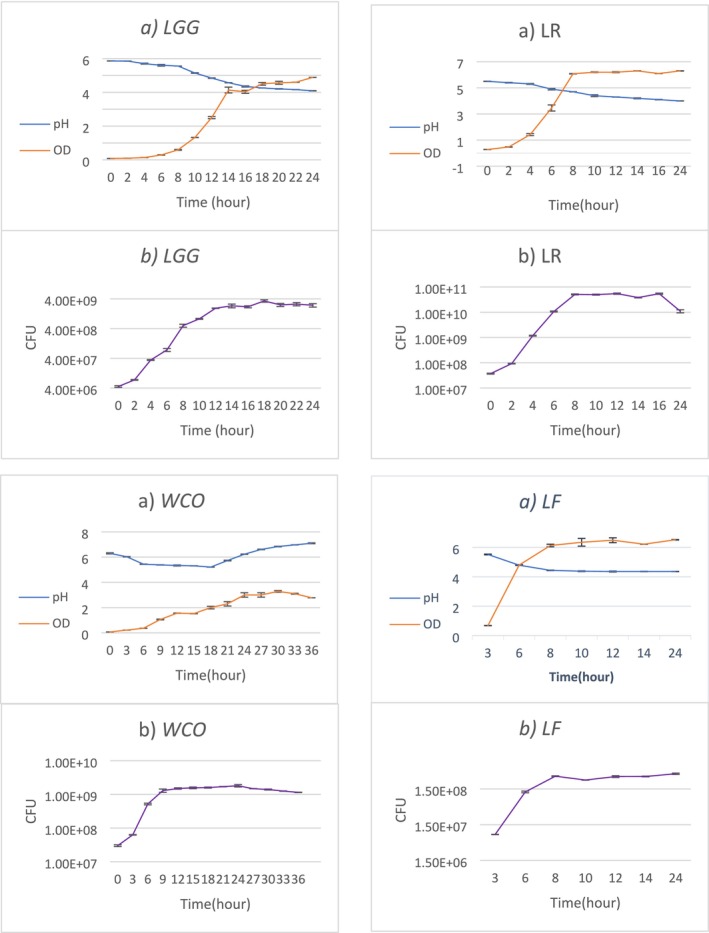
Growth characteristics of *Lactobacillus rhamnosus* (*LGG*), *Limosilactobacillus* reuteri (*LR*), *Limosilactobacillus fermentum* (*LF*), and *Weizmannia coagulans* (*WCO*). (a) Changes in optical density (OD_600_) and pH over time during incubation. (b) Changes in viable cell counts (CFU/mL) over time. Data are presented as mean ± standard errors (SE) from three independent biological replicates.

The growth kinetics of *LGG*, *LR*, *LF*, and *WCO* were analyzed to optimize postbiotic production. As shown in Figure 2, growth curves, pH dynamics, and colony‐forming unit (CFU) revealed distinct strain‐specific profiles. *LGG* exhibited a 4‐h lag phase, followed by an 8‐h exponential growth phase, before entering the stationary phase at 12 h. In contrast, *LR* and *WCO* displayed shorter lag phases (2 h) and finished exponential growth phases (6 h), reaching stationary phases at 8 and 14 h, respectively. Notably, *LF* demonstrated biphasic growth, with a second exponential phase emerging after 6 h, suggesting metabolic adaptability or nutrient scavenging under the tested conditions (Figure 2a).

The pH of the culture medium decreased from an initial 6.5 to 4.2–4.5 during exponential growth, consistent with lactic acid production characteristic of LAB metabolism. This acidification correlated with an increase in optical density (OD_600_) and CFU counts (Figure 2b), confirming active biomass accumulation. *LR* achieved the highest cell density (1.2 × 10^9^ CFU/mL at 8 h), followed by *LGG* (9.5 × 10^8^ CFU/mL at 12 h), while *LF* showed delayed growth but reached comparable final biomass (8.8 × 10^8^ CFU/mL at 18 h). These strain‐dependent growth patterns align with previous reports on LAB fermentation kinetics [22].

These findings underscore the importance of tailoring fermentation protocols to individual strain kinetics to maximize both postbiotic yield and quality. The subsequent sections investigate how these distinct growth profiles correlate with the observed antibacterial and antioxidant activities.

### 3.2 Bactericidal Effects of CFS From Lactic Acid Bacteria

CFS derived from LAB exhibited significant antimicrobial activity against *M. luteus*, *S. aureus* , and *E. coli* (Table 1). Among them, CFS of *LGG* demonstrated the strongest overall inhibitory effect. It was particularly potent against *M. luteus* , producing a zone of inhibition (ZOI) of 30 ± 1 mm and demonstrating the lowest MIC/MBC of 5% and 7.5% v/v, respectively. Although active against the other pathogens, its effect was less pronounced, with ZOIs of 20 ± 1 mm against *S. aureus* and 17 ± 2 mm against *E. coli* .

**TABLE 1:** Antibacterial activity of lactic acid bacteria strains against *Micrococcus luteus* , *Staphylococcus aureus* , and *Escherichia coli* determined by the well diffusion assay and minimum bactericidal/inhibitory concentration method. The inhibition zone results are presented as mean ± standard deviation (SD). MIC and MBC values are presented as mean percentages obtained from three independent biological replicates.

	IZD (mm)	MIC (%)	MBC (%)
*M. luteus*			
CFS of *LGG*	30 ± 1	5	7.5
CFS of *LR*	30 ± 0.5	6.25	10
CFS of *LF*	30 ± 0.2	6.25	10
CFS of *WCO*	25 ± 1	12.5	15
*E. coli*			
CFS of *LGG*	17 ± 2	3.125	3.75
CFS of *LR*	15 ± 1	7.5	20
CFS of *LF*	15 ± 1	5	10
CFS of *WCO*	10 ± 0.5	7.5	15
*S. aureus*			
CFS of *LGG*	20 ± 1	3.125	7.5
CFS of *LR*	17 ± 1	5	7.5
CFS of *LF*	18 ± 1	3.125	5
CFS of *WCO*	14 ± 1	7.5	15

The CFS from *LR* and *LF* also showed strong activity against *M. luteus* (ZOI≈30 mm), but their higher MIC and MBC values (6.25% and 10% v/v, respectively) indicated lower potency compared to *LGG*. In contrast, *WCO* demonstrated the weakest activity against this bacterium (ZOI = 25 ± 1 mm; MIC = 12.5% v/v; MBC = 15% v/v).

Against Gram‐negative *E. coli* , all LAB strains showed moderate inhibition (ZOI = 10–17 mm), with *LGG* again exhibiting the lowest MIC and MBC values (3.125% and 3.75% v/v). Similar trends were observed against *S. aureus* , where both *LGG* and *LF* showed the most potent inhibition with MIC values of 3.125% v/v.

### 3.3 Evaluation of Antioxidant Activity: DPPH Radical Scavenging

The antioxidant capacity of CFS from LAB was evaluated using the 1,1‐diphenyl‐2‐picrylhydrazyl (DPPH) radical scavenging assay. Among the tested strains, *WCO* exhibited the most potent antioxidant activity, achieving an IC_50_ value of 17.48 μg/mL the lowest of all LAB strains and comparable to the reference antioxidant ascorbic acid (IC_50_ = 32.50 μg/mL) (Table 2). *LGG* and *LR* demonstrated moderate antioxidant activity, with IC_50_ values of 22.16 and 20.99 μg/mL, respectively. *LF* showed the weakest activity (IC_50_ = 36.48 μg/mL). Concentration‐dependent scavenging was observed across all strains, with *WCO* achieving 84.48% inhibition at 90 μg/mL and 33.04% at 10 μg/mL. Notably, *LGG* exhibited high scavenging activity at elevated concentrations (94.73% at 80 μg/mL), while *LR* reached 98.94% inhibition at 100 μg/mL.

**TABLE 2:** Antioxidant activity of lactic acid bacteria cell‐free supernatants evaluated by the DPPH radical scavenging assay. The percentage of DPPH inhibition was measured at different CFS concentrations, and IC_50_ values were calculated accordingly. Data are presented from three independent biological replicates. The inhibition zone results are presented as mean ± standard deviation (SD).

Sample	Concentration	Inhibition (%)	IC_50_
*WCO*	90	84.48% ± 2.1	17.48 ± 0.82
	80	77.58% ± 3.22	
	30	66.85% ± 1.32	
	20	55.55% ± 1.51	
	10	33.04% ± 0.91	
*LF*	80	73.18% ± 3.3	36.48 ± 2.36
	40	59.86% ± 0.52	
	30	38.98% ± 1.1	
	20	31.70% ± 2.54	
	10	1.53% ± 0.01	
*LGG*	80	94.73% ± 098	22.16 ± 1.07
	60	91.95% ± 1.3	
	50	86.97% ± 0.88	
	40	74.37% ± 2.31	
	30	66.66% ± 3.3	
	20	43.48% ± 2.1	
	10	16.85% ± 1.43	
*LR*	100	98.94% ± 2.25	20.99 ± 0.52
	90	83.14% ± 2.31	
	30	46.45% ± 2.19	
	10	40.99% ± 0.22	
Ascorbic acid	100	94.45% ± 0.38	32.50 ± 0.36
	50	78.92% ± 1.81	
	25	31.22% ± 0.24	

### 3.4 Characterization of Hyalurosomes

#### 3.4.1 Hyalurosome Morphology

**FIGURE 3:**
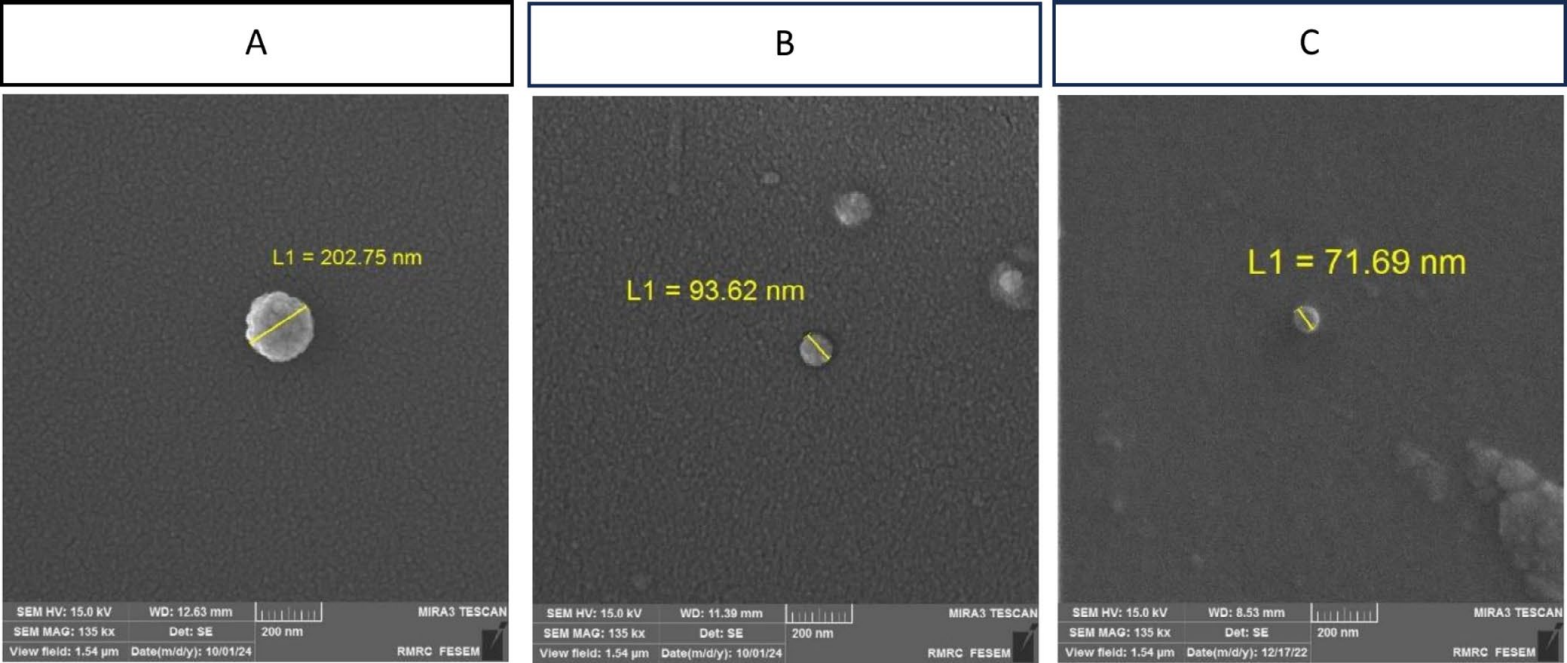
Scanning electron microscopy (SEM) images of hyalurosomes. (A) *WCO‐hyalurosomes*, (B) LGG‐hyalurosomes, and (C) unloaded hyalurosomes (control). Samples were dried and sputter‐coated with a thin layer of gold prior to imaging. Images were acquired using a SEM instrument at an accelerating voltage of 15 kV and a magnification of ×50 000. Scale bar: 200 nm. The images show the spherical morphology and nanometric size of the prepared hyalurosomes.

The morphology of hyalurosomes, both unloaded and *WCO/LGG* loaded, was characterized using scanning electron microscopy (SEM). As shown in Figure 3, all hyalurosomes exhibited a uniform spherical morphology with smooth surfaces. The unloaded hyalurosomes (Figure 3C) and those loaded with *WCO* (Figure 3A) or LGG (Figure 3B) displayed comparable structural integrity, confirming that postbiotic encapsulation did not alter vesicle shape.

Particle sizes ranged from 70 to 200 nm, consistent with dynamic light scattering (DLS) measurements reported in Table 3. This narrow size distribution, combined with the spherical morphology, suggests favorable colloidal stability and suitability for topical delivery. The ζ potential measurements (Table 3) further corroborated the stability of the formulations, with surface charges ranging from −6.96 to −50.95 mV, indicative of electrostatic repulsion preventing aggregation.

**TABLE 3:** Physicochemical properties of hyalurosomes.

Nano particle	Vesicle size (nm)	PDI	Surface charge (mv)
hyalurosome	100–120	0.415	−50.95
*LGG‐*hyalurosome	80–160	0.430	−6.96
*WCO‐*hyalurosome	85–195	0.551	−19.49

*Note:* Mean vesicle size, polydispersity index (PDI), and zeta potential of hyalurosomes are presented. Measurements were performed using dynamic light scattering (DLS). Vesicle size and PDI indicate the uniformity of the colloidal dispersion, while zeta potential reflects the surface charge and colloidal stability of the formulations.

#### 3.4.2 Particle Size and ζ Potential

The physicochemical properties of unloaded and postbiotic‐loaded hyalurosomes, including hydrodynamic size, polydispersity index (PDI), and ζ potential, are summarized in Table 3. Unloaded hyalurosomes exhibited a narrow size distribution (100–120 nm) with a PDI of 0.415, indicating high monodispersity. Loading with LGG or *WCO* increased the vesicle size range to 80–160 nm and 85–195 nm, respectively, reflecting successful encapsulation of bioactive compounds. The PDI values remained below 0.7 for all formulations (0.430 for LGG‐loaded and 0.551 for *WCO*‐loaded), confirming acceptable colloidal stability.

The ζ potential of unloaded hyalurosomes was highly negative (−50.95 mV), attributed to the anionic carboxyl groups of hyaluronic acid. Postbiotic encapsulation reduced the surface charge to −6.96 mV (LGG‐loaded) and −19.49 mV (*WCO*‐loaded), likely due to electrostatic interactions between the negatively charged HA and cationic components of the postbiotics. Despite this reduction, the moderate ζ potential values (>|−20 mV|) suggest sufficient electrostatic repulsion to prevent aggregation during storage.

#### 3.4.3 Evaluation of Release Kinetics of *LGG* ‐Hyalurosome by Normal Saline Test (CFU Assay Was Used)

The sustained antibacterial efficacy of *LGG*‐hyalurosomes was evaluated using a colony‐forming unit (CFU) assay in normal saline. Unloaded hyalurosomes, which inherently lacked antibacterial activity, served as a negative control, while saline containing *M. luteus* or *S. aureus* alone constituted the positive control. Initial assessments confirmed that the postbiotic derived from *LGG* exhibited the highest antibacterial activity among the tested strains. Integration of this postbiotic into hyalurosomes—nanoparticles initially devoid of antibacterial properties—resulted in a marked reduction of pathogenic colonies, providing direct evidence of successful encapsulation.

At 24 h, the number of *M. luteus* colonies in the presence of *LGG*‐hyalurosomes decreased to 30 ± 1 CFU/mL, a 97.5% reduction compared to unloaded hyalurosomes (1200 ± 28.87 CFU/mL) and the positive control (1216.67 ± 57.74 CFU/mL). Similarly, *S. aureus* colonies declined to 1233.33 ± 7.09 CFU/mL with LGG‐hyalurosomes, contrasting sharply with the unloaded hyalurosomes (1100 ± 100 CFU/mL) and positive control (1100 ± 100 CFU/mL). By 48 h, complete eradication of both pathogens was observed (0 CFU/mL for LGG‐hyalurosomes), while unloaded hyalurosomes and controls retained significant bacterial viability (Table 4).

**TABLE 4:** Antibacterial activity of *LGG*‐hyalurosome assessed by the normal saline method. Results are expressed as CFU/mL after 24 and 48 h incubation at 37°C (mean ± SD; *n* = 3).

Sample	24 h (CFU/mL)	SD	48 h (CFU/mL)	SD
*LGG‐h*yalurosome	30	1	0	0
Hyalurosome	1200	28.86751	1016.667	76.37626
*S. aureus*	1216.667	57.73503	1100	100
*LGG‐h*yalurosome	1233.333	7.094599	0	0
Hyalurosome	46.33333	28.86751	543.3333	50.33223
*M. luteus*	433.3333	28.86751	713.3333	32.1455

#### 3.4.4 Evaluation of Encapsulation of *WCO‐*Hyalurosome by DPPH Assay

The encapsulation efficiency of *WCO* within hyalurosomes was evaluated using the DPPH radical scavenging assay. Unloaded hyalurosomes alone exhibited negligible antioxidant activity, confirming the absence of inherent radical‐scavenging properties. In contrast, *WCO*‐hyalurosomes demonstrated significant DPPH radical inhibition, with an IC_50_ value of 37.63 μg/mL.

The time‐dependent stability of the encapsulated postbiotics was further validated by measuring DPPH scavenging activity at 0, 24, and 48 h. No significant degradation in antioxidant efficacy was observed over this period, underscoring the protective role of hyalurosomes in preserving bioactive integrity.

### 3.5 MTT Assay for Cytotoxicity Evaluation

The cytotoxicity of free and encapsulated postbiotics derived from LGG and *WCO* was evaluated on human dermal fibroblasts using the MTT assay. LGG and *WCO* at a 50% (v/v) concentration reduced fibroblast viability to approximately 60%, indicating moderate cytotoxicity. However, encapsulation of these postbiotics within hyalurosomes significantly improved biocompatibility, with cell viability increasing to over 90% at the same concentration.

**FIGURE 4:**
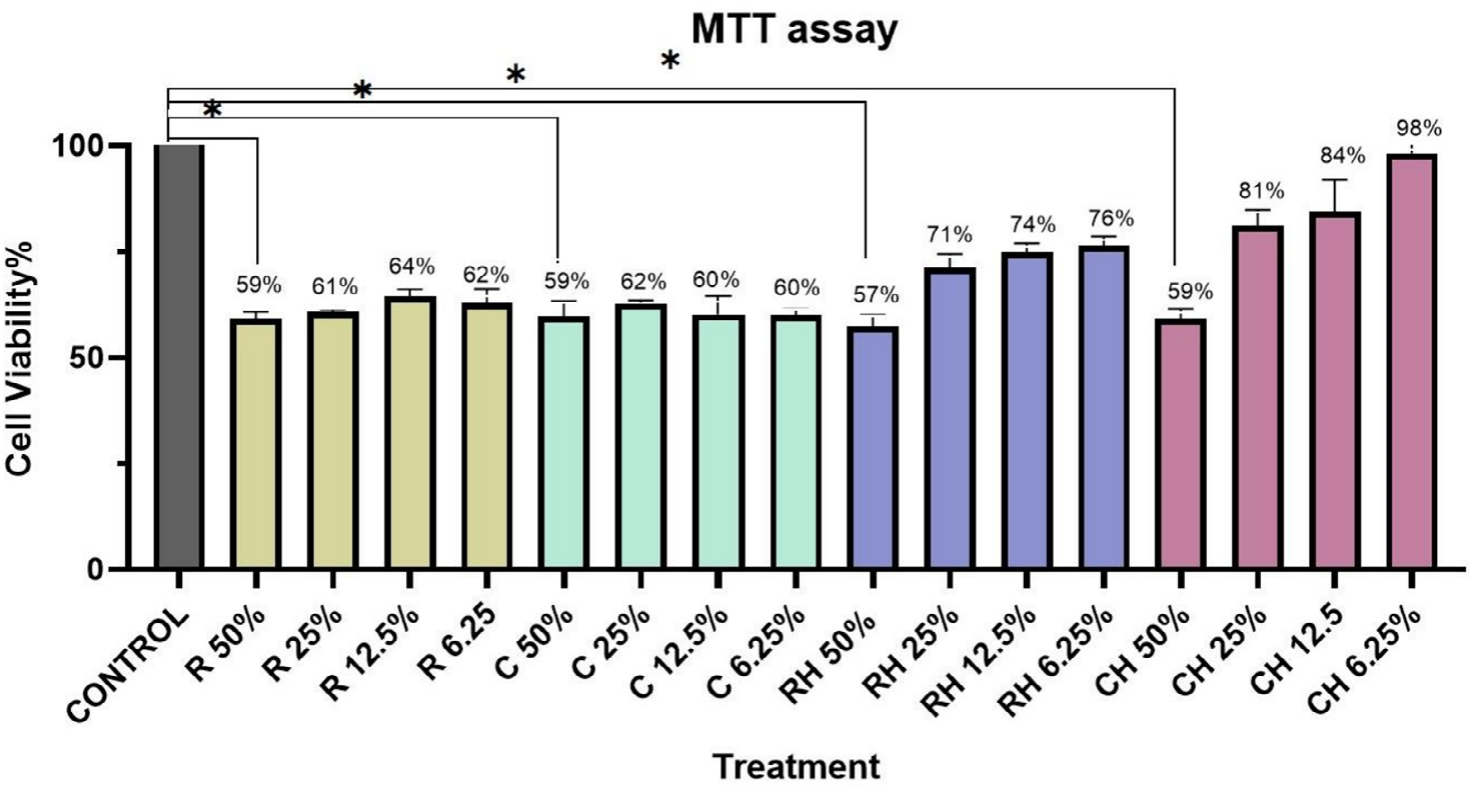
Cytotoxicity results on human normal fibroblast cells, analyzed by ANOVA, are presented. In this figure, ‘R' represents 10‐fold CFS of *LGG*, added to cells at four concentrations: 50%, 25%, 12.5%, and 6.25%. Similarly, ‘C' represents 10‐fold CFS of *WCO*, ‘RH’ represents 10‐fold LGG‐hyalurosome, and ‘CH’ represents 10‐fold WCO‐hyalurosomes. The asterisk (*) indicates a statistically significant difference compared to the control group (*p* < 0.05).

A concentration‐dependent reduction in cytotoxicity was observed for encapsulated postbiotics. At a 6.25% (v/v) dilution, viability remained above 90% for both LGG‐ and *WCO*‐hyalurosomes, compared to 60% viability for their free counterparts (Figure 4). Unloaded hyalurosomes exhibited no cytotoxicity, confirming the inherent safety of the nanocarrier itself.

## 4 Discussion

This study demonstrates that postbiotics from select LAB strains possess significant antimicrobial and antioxidant activities. Furthermore, we show that encapsulation in HA‐based hyalurosomes can enhance their delivery and biocompatibility, addressing key challenges for topical application.

The growth kinetics of the four LAB strains revealed distinct profiles with direct implications for postbiotic production. *WCO* and *LR*, with their short lag phase, appear suitable for rapid, high‐yield fermentation cycles. In contrast, the biphasic growth of *LF* suggests metabolic adaptability, potentially leading to a unique profile of bioactive metabolites that warrants further investigation. These strain‐specific patterns underscore the importance of tailoring fermentation protocols to maximize the yield and functionality of postbiotic compounds.

Among the strains, *LGG* postbiotics demonstrated superior antibacterial activity. The potent efficacy against *M. luteus* (ZOI = 30 ± 1 mm, MIC/MBC = 5/7.5% v/v), coupled with strong activity against *S. aureus* and *E. coli* , highlights its broad‐spectrum potential. The low MIC values (≤ 3.125% v/v for *S. aureus* ) are particularly promising for targeting skin pathogens, including potentially antibiotic‐resistant strains. This activity is likely attributable to a combination of antimicrobial metabolites, such as bacteriocins and organic acids, which are well‐documented in *Lactobacillus* species.

Conversely, *WCO* postbiotics exhibited exceptional antioxidant capacity, showing an IC_50_ = 17.48 μg/mL that was superior to ascorbic acid in the DPPH assay. This potent, concentration‐dependent scavenging activity suggests *WCO* as a promising natural alternative to synthetic antioxidants for combating oxidative stress in skin. Crucially, this antioxidant potency was largely retained after encapsulation, confirming that the hyalurosome fabrication process did not degrade the key bioactive compounds.

The robust bioactivity observed is underpinned by the diverse mechanisms of action of postbiotic metabolites. The antibacterial effects are likely mediated not only by organic acids (e.g., lactic and acetic acid) that disrupt microbial membrane integrity and intracellular pH, but also by bacteriocins and other antimicrobial peptides that target specific cell wall components. Similarly, the potent antioxidant activity can be attributed to a suite of metabolites, including extracellular proteins, peptides, and biosurfactants produced by LAB, which can act as direct free radical scavengers and metal chelators. This multi‐mechanistic profile enhances their therapeutic potential and reduces the likelihood of resistance development.

The choice of an appropriate delivery system was critical to translating this bioactivity. While conventional liposomes are widely used, they can suffer from instability and limited skin penetration [23]. More advanced vesicles like ethosomes, which utilize high ethanol concentrations for enhanced permeation, may raise toxicity concerns for sensitive skin [24]. Transfersomes, known for their ultradeformability, are highly effective penetrators but can be less stable during storage [25]. In this context, hyalurosomes offer a unique combination of advantages: the intrinsic biocompatibility, hygroscopicity, and CD44‐receptor targeting capability of HA [18], combined with a structurally robust vesicular system. Our characterization confirmed the successful formation of monodisperse, spherical hyalurosomes (70–200 nm) with a negative surface charge, ideal for colloidal stability. The sustained antibacterial release from *LGG*‐hyalurosomes, eradicating *M. luteus* and *S. aureus* within 48 h, demonstrates the capability of this system to provide prolonged activity at the target site. The absence of antibacterial activity in unloaded hyalurosomes further validates the successful encapsulation and functional retention of *LGG* within the nanoparticles. Evaluation of encapsulation of *WCO*‐hyalurosome by DPPH assay results confirms that the encapsulation process did not compromise the functional properties of *WCO*, thereby establishing hyalurosomes as a robust delivery system for antioxidant agents in topical applications.

Furthermore, the hyalurosomes markedly improved the biocompatibility of the postbiotics. While free postbiotics reduced human dermal fibroblast viability to 60% at high concentrations, encapsulation restored viability to over 90%. These results demonstrate that hyalurosomes effectively mitigate the cytotoxic effects of postbiotics while maintaining their bioactive delivery. This mitigation of cytotoxicity, paired with the inert nature of the unloaded hyalurosome carrier, is a significant advantage for chronic topical use in cosmetic and therapeutic formulations, where minimal irritancy is paramount.

While this study provides a robust in vitro proof of concept, it has limitations. Translational applications require in vivo validation in models of skin infection or photoaging. Furthermore, the long‐term stability of the hyalurosomes under storage conditions and the scalability of their production warrant further investigation. Future work should also explore the potential synergistic effects of multi‐strain postbiotic combinations.

In conclusion, this work highlights the potential of LAB‐derived postbiotics as multifunctional skincare agents. By integrating them into HA‐based hyalurosomes, we have developed a delivery system that enhances their stability, provides sustained release, and significantly improves their safety profile. This strategy represents a promising, nanotechnology‐driven approach to addressing the interconnected challenges of skin microbiota dysbiosis and oxidative stress.

## 5 Conclusions

This study comprehensively evaluated the bioactive potential of postbiotics derived from *Limosilactobacillus reuteri* ATCC 23272, *Lacticaseibacillus rhamnosus* ATCC 53103 (*LGG*), *Limosilactobacillus fermentum* ATCC 9338, and *Weizmannia coagulans* ATCC 5856 (*WCO*), with a focus on their antibacterial and antioxidant properties. Among these, *LGG* demonstrated superior antibacterial efficacy, exhibiting a 30 mm inhibition zone against *M. luteus* and achieving bactericidal activity at minimal concentrations (MIC = 5%, MBC = 7.5% v/v). *WCO* emerged as the most potent antioxidant, with an IC_50_ of 17.48 μg/mL in the DPPH assay.

The encapsulation of *LGG* and *WCO* within hyalurosomes yielded nanoparticles with optimal physicochemical characteristics: spherical morphology, hydrodynamic sizes of 80–200 nm, and negative surface charges (−6.96 to −19.49 mV). The low polydispersity indices (PDI < 0.7) confirmed monodisperse distributions. Notably, the encapsulated postbiotics retained their bioactivity, with *LGG*‐hyalurosomes achieving complete eradication of *M. luteus* and *S. aureus* within 48 h, while *WCO*‐hyalurosomes preserved 84.48% DPPH radical scavenging activity at 90 μg/mL.

Crucially, hyalurosomes significantly reduced cytotoxicity compared to free postbiotics, enhancing human dermal fibroblast viability from 60% to over 90% at equivalent concentrations.

These findings establish LAB‐derived postbiotics, particularly when delivered via hyalurosomes, as promising candidates for advanced skincare formulations. Future studies should explore in vivo efficacy, long‐term stability, and synergistic effects of multi‐strain postbiotics to accelerate clinical translation.

## Author Contributions

**Seyede Fateme Morakabi:** served as the primary investigator, carried out 80% of the experiments, and drafted the manuscript. **Mahvash Khodabandeh Shahraky:** conceptualized the study, designed the experiments, and provided overall guidance. **Gholamreza Ahmadian:** provided a critical review and editing of the manuscript. **Roghayeh Mokhtari Asl:** developed and implemented the computational models and provided technical support for data analysis. All authors have read and approved the final version of the manuscript.

## Ethics Statement

The authors have nothing to report.

## Consent

The authors have nothing to report.

## Conflicts of Interest

The authors declare no conflicts of interest.

## Data Availability

The data that support the findings of this study are available from the corresponding author upon reasonable request.
